# The Effects of Interventions with Glucosinolates and Their Metabolites in Cruciferous Vegetables on Inflammatory Bowel Disease: A Review

**DOI:** 10.3390/foods13213507

**Published:** 2024-11-01

**Authors:** Jichun Zhao, Xiaoqin Zhang, Fuhua Li, Xiaojuan Lei, Lihong Ge, Honghai Li, Nan Zhao, Jian Ming

**Affiliations:** 1 College of Food Science, Southwest University, Chongqing 400715, China; jichunzhao@swu.edu.cn (J.Z.);; 2 Chongqing Key Laboratory of Speciality Food Co-Built by Sichuan and Chongqing, Chongqing 400715, China; 3 Research Center for Fruits and Vegetables Logistics Preservation and Nutritional Quality Control, Southwest University, Chongqing 400715, China; 4 College of Life Science, Sichuan Normal University, Chengdu 610101, China; 5 Institute of Agro-products Processing Science and Technology, Sichuan Academy of Agricultural Sciences, Chengdu 610066, China

**Keywords:** IBD, glucosinolates, cruciferous vegetables, gut microbiota, antioxidant, anti-inflammatory

## Abstract

Inflammatory bowel disease (IBD) is a chronic inflammatory disorder of the gastrointestinal tract which affects millions of individuals worldwide. Despite advancements in treatment options, there is increasing interest in exploring natural interventions with minimal side effects. Cruciferous vegetables, such as broccoli, cabbage, and radishes, contain bioactive compounds known as glucosinolates (GLSs), which have shown promising effects in alleviating IBD symptoms. This review aims to provide a comprehensive overview of the physiological functions and mechanisms of cruciferous GLSs and their metabolites in the context of IBD. Reviewed studies demonstrated that GLSs attenuated all aspects of IBD, including regulating the intestinal microbiota composition, exerting antioxidant and anti-inflammatory effects, restoring intestinal barrier function, and regulating epigenetic mechanisms. In addition, a few interventions with GLS supplementation in clinical studies were also discussed. However, there are still several challenges and remaining knowledge gaps, including variations in animals’ experimental outcomes, the bioavailability of certain compounds, and few clinical trials to validate their effectiveness in human subjects. Addressing these issues will contribute to a better understanding of the therapeutic potential of cruciferous GLSs and their metabolites in the management of IBD.

## 1. Introduction

Inflammatory bowel disease (IBD) is a non-specific bowel disorder characterized by inflammation and disruption of the intestinal epithelial barrier, mainly including ulcerative colitis (UC) and Crohn’s disease (CD) [1]. The prevalence of IBD in developing countries was relatively low compared with North America and western European countries, but the incidence of IBD has been rising rapidly in newly industrialized countries in South America, Asia, and Africa in the past few decades due to their westernized lives [2,3,4]. IBD has been a public health challenge worldwide [5].

The pathophysiology of IBD is attributed to a complicated combination of genetic, microbial and environmental factors involving aberrant immunological responses to the intestinal microbiota, leading to chronic intestinal inflammation [6]. IBD not only damages the intestinal epithelial structure but also triggers more diseases through the gut-organ axis [7]. Epidemiological studies demonstrated that IBD patients have a higher risk of Alzheimer’s disease (AD), a neurodegenerative condition, and both diseases showed that the intestinal microbiota and metabolites changed in a similar way, suggesting that IBD may be one of the potential pathogenic factors of AD through bidirectional communication between intestinal microbes and the brain [8]. Clinically, drugs such as sulfasalazine, glucocorticoids, thioguanins and thiopurine can alleviate IBD symptoms. However, these medications do not alter the overall course of IBD and have many adverse effects, including gastrointestinal complaints (diarrhea, vomiting, abdominal pain, etc.), hepatotoxicity, pancreatitis and hyperglycemia [9]. Older adults are more susceptible to these adverse events than younger people [6].

Dietary therapy plays a critical part in IBD management, from prevention of the disease to active disease and complication treatment. Several diets have been investigated to treat active IBD, including the specific carbohydrate diet, Mediterranean diet, plant-based diet and low-emulsifier diet [10]. Moreover, studies have shown that dietary components can benefit the treatment of intestinal diseases at different levels through inhibiting intestinal inflammation, restoring gut microbiota and renewed intestinal epithelial and improved barrier function [11,12]. Dietary intakes of fruit and vegetables are correlated with a reduced risk of developing IBD (UC and CD) and are recommended for prevention of IBD [13,14]. Among them, cruciferous vegetables such as broccoli, cabbage and cauliflower are part of a balanced diet and rich in vitamins, minerals and dietary fiber as well as phytochemicals such as glucosinolates (GLSs). GLSs are a kind of secondary metabolite of cruciferous vegetables, which have attracted increasing attention recently. GLSs and their metabolites have anti-inflammatory, anticarcinogenic, antibacterial, antioxidant and other activities, and they also exert protective effects on IBD in mouse models and human intestinal cell models [15].

In this review, the metabolism in vivo and physiological functions of cruciferous GLSs are summarized, mainly focusing on the protective mechanism of GLSs and their metabolites on IBD, including gut microbiota modulation, antioxidant activity, anti-inflammatory activity and maintaining the intestinal barrier. Furthermore, the influences of processing methods on the content of total GLSs and isothiocyanates (ITCs) are also reviewed to provide perspectives and foundations for dietary prevention of IBD in the future.

## 2. Cruciferous Vegetables and Glucosinolates

Cruciferous vegetables, characterized by their four equal-sized petals [12], belong to the family *Brassicaceae* and include widely consumed vegetables such as broccoli, kale, mustard, radish and cabbage. These cruciferous vegetables possess a variety of health-promoting activities, which are ascribed to their secondary metabolites [16]. Of them, GLSs are a group of nitrogen- and sulfur-containing water-soluble secondary metabolites, which are synthesized by amino acid (especially methionine) chain extension, formation of the glucosinolate core structure and the secondary modification of side chains [17]. Therefore, all GLS compounds are structurally similar, including β-D-thioglucoside groups, sulfonated moieties and R groups of amino acid side chains of different origins [18]. According to the different R groups, it can be divided into aliphatic groups (side chains derived from methionine, alanine, valine, leucine and isoleucine, among other amino acids), aromatic (side chains derived from tyrosine and phenylalanine) and indole groups (side chains derived from tryptophan), of which aliphatic types account for the largest proportion [19].

The GLS content varies with cruciferous vegetables varieties, growth environments, different organs, and signal substances which induce responses [19,20]. For kale, broccoli, mustard and Chinese cabbage, their GLSs are mainly aliphatic and indole individuals (Table 1). There are differences in the types and amounts of GLSs among cruciferous vegetables. Variation in GLS content has also been observed among different organs in the same vegetable [19]. Therefore, the total GLS content of vegetables falls within a certain range, with the highest content found in seeds. In addition to the edible parts of vegetables, their roots and leaves are also rich in GLSs, and thus these parts can be reasonably utilized for their contained GLSs, aside from being used as feed or fertilizer or discarded as waste [19].

## 3. Physiological Functions of GLSs and Metabolites

### 3.1. Metabolism of GLSs In Vivo

In intact plants, GLSs are chemically stable, biologically inert and not highly active, but they can be degraded by myrosinase and influenced by environmental factors such as the temperature, light and pH [23]. GLSs and myrosinase are stored in different cells of cruciferous vegetables. Once the plant tissues are disrupted, they meet, react and produce ITCs, nitriles, epithionitriles (ETNs), etc.

When cruciferous vegetables are chewed, enzymatic degradation also occurs in the human gastrointestinal tract [24]. Thirteen GLS metabolites were found in human urine and plasma after broccoli consumption, including sulforaphane (SFN), sulforaphane-cysteine and sulforaphane-*N*-acetyl-cysteine from glucoraphanin (GRP), indole-3-carbinol (I3C), indole-3-carboxaldehyde and indole-3-carboxylic acid from glucobrassicin (GBS) and methoxyl indole-3-carbinol, methoxyl-indole-3-carboxaldehyde, methoxyl-indole-3-carboxylic acid and methoxyl-ascorbigen from methoxyl glucosinolates [25]. Oral administration of broccoli leaves demonstrated that low levels of GLSs were absorbed in mice. Only glucoerucin (GER), GBS, SFN and I3C were detected in plasma, and SFN and its derivatives mainly remained in the intestinal tract and then appeared in the feces [26].

The metabolism of GLSs in vivo is attributed to myrosinase derived from plant tissue or the human microbiota. Since the first bacterial myrosinase was detected, a large number of microorganisms which can metabolize GLSs have been isolated [24], including *Lactobacillus*, *Bifidobacterium*, *Bacteroides*, *Enterococcus* and *Escherichia coli*, which inhabit different parts of the mammalian intestine [27,28], as well as *Aspergillus niger*, *Aspergillus syndowi* and other fungi [23,29], but these bacterial myrosinases have lower hydrolyzing efficiencies than that of plant myrosinase, and the main metabolite of glucorabhanin is nitrile rather than SFN [30]. Lastly, consumption of cruciferous vegetables can induce myrosinase-like activity in intestinal microbiota. For example, four days of broccoli consumption enhanced the conversion of GRP to the corresponding ITCs by the cecal microbiota ex vivo [31].

### 3.2. Alleviating Effects of GLSs and Their Metabolites on Colitis

GLS metabolites possess several health benefits, such as antioxidant, antibacterial, anticancer and anti-inflammatory activities as well as regulation of intestinal flora and other effects [28,32,33]. The physiological functions of GLSs and their metabolites have been discussed in previous reviews [12,16]. Our review mainly focuses on the alleviating effects of GLSs and their metabolites on IBD.

GLSs and their metabolites are promising in the prevention of gastrointestinal diseases. For example, phenylethyl isothiocyanate (PEITC) exerts anti-inflammatory activities by inhibiting NF-κB activation in gastrointestinal inflammation diseases [34]. Benzyl isothiocyanate (BITC) can regulate inflammation, oxidative stress, and apoptosis through the nuclear factor erythroid 2-related factor 2 (Nrf2)/heme oxygenase-1 (HO-1) and NF-kB signaling pathways, thus exerting anti-gastric ulcer effects [35].

Previous studies have found that cruciferous vegetables, such as GLS-rich broccoli, cabbage and mustard greens, can effectively alleviate the symptoms of IBD in human intestinal cell models and nematode and murine models (Table 2). For intestinal colitis study, dextran sodium sulfate (DSS, 2~5%)-induced mice colitis is one of the most widely used models [36], characterized by extensive damage to the intestinal epithelium, depletion of goblet cells, infiltration of inflammatory cells, and the presence of edema [37,38].

Cruciferous vegetables, GLSs and metabolites have demonstrated a potent effect in attenuating DSS-induced colitis symptoms. Both raw broccoli and lightly cooked broccoli can alleviate the DSS-induced disease activity index (DAI), relieve symptoms such as a 50% reduction body weight, stool bleeding, a shortened colon length and increased intestinal barrier permeability, and reduce colonic injury [15,39]. GRP supplementation improved body weights, stool blood, fecal consistency, and colon tissue morphology in DSS-induced colitis mice [40]. SFN can reverse body weight loss and shortened colon lengths, improve intestinal gland damage and bleeding and reduce the fibrotic area in colitis models [29,41,42]. I3C treatment in 2,4,6-trinitrobenzenesulfonic acid (TNBS)-induced colitis mice maintained crypt formation and a normal colon tissue structure, and it showed signs of reduced cellular infiltration [43]. Meanwhile, 3,3′-Diindolylmethane (DIM), produced by the digestion of I3C, can repair IL-1β-induced differentiation of the human intestinal Caco-2 cell monolayer barrier and restore its permeability [44]. Allyl isothiocyanate (AITC)-treated intestinal epithelium showed a relatively intact structure and more goblet cells [45]. However, to our knowledge, there are few clinical trials for GLS and isothiocyanates and their effect on intestinal inflammation.

**Table 2:** Effect of cruciferous vegetables, GLSs and metabolites on alleviating the symptoms of IBD.

Models	Inducement	Intervention	Effects	References
C57BL6/J-*Ahr*^b/b^ and *Ahr*^d/d^ mice, 8–10 weeks	drinking 3.5% DSS water for 6 days	15% broccoli diet for 14 days beforehand	broccoli diet significantly attenuated the clinical manifestation of splenomegaly and DAI in *Ahr*^b/b^ and *Ahr*^d/d^ mice	[46]
C57BL/6 mice, male, 8–10 weeks	drinking 2.5% DSS for one week	10% raw broccoli (RB), 10% lightly cooked broccoli (CB)	both CB and RB effectively reduced DAI, extended colon length and induced less blood endotoxin and less severe colon lesions	[39]
interleukin (IL)-10-knockout mice on C57BL/6 background	inoculation with *Helicobacter hepaticus*	diet with 10% raw broccoli sprouts	broccoli sprout diet reduced weight stagnation, fecal blood and diarrhea, enhanced gut microbiota richness and reduced the prevalence and abundance of pathobiont bacteria triggering inflammation	[47]
C57BL/6J mice, female and male	drinking 2% DSS water for 5 days for SPF mice and 1% DSS for GF mice	5% steamed broccoli sprout (SBS) diet for 4–6 weeks	SBS decreased DSS-induced colitis via the gut microbiota converting cruciferous vegetables into bioactive metabolites, promoting anti-inflammatory effects	[15]
C57BL/6J mice. male and female, 7–8 weeks	aater with 3% DSS for 1 week, followed 1 week of recovery and then 1 week 3% DSS	red cabbage juice (RCJ)	RCJ significantly improved body weight and survival of mice, decreased DAI scores, improved intestinal barrier integrity by enhancing the expression of colonic mucins and tight junction (TJ) proteins and the abundance of SCFA-producing bacteria and increased PPAR-γ activation	[48]
Wistar rats, male	drinking 4% DSS water for 6 days, mild colitis	diet with 8750 mg/kg broccoli sprout extract (BSE)	diets with BE reduced the DSS-induced rise in the expression of pro-inflammatory mediators NFκB, MCP-1, COX2 and VCAM-1	[49]
C57BL/6J mice male, 6 weeks old	25 g/L DSS water	370 mg/kg·day BSE dissolved in 0.2 mL of skim milk	BSE administration increased body weight, improved antioxidant activities and restored the intestinal barrier through enhancing TJ protein expression	[50]
C57BL/6J mice, male, 7 weeks	drinking 3% DSS water for 5 days (acute UC) and cyclic rotations of 2.5% DSS water for 30 d	moringa seed extract (MSE)	MSE decreased DAI scores and colon weight/length ratios, increased colon lengths, reduced colonic inflammation and damage in acute UC, decreased colonic pro-inflammatory expression and downregulated gene expression of pro-inflammatory activity	[51]
Sprague Dawley rats, male, 3 months	intrarectal injection with 2,4-dinitrobenzenesulfonic acid (DNBS, 20 mg in 0.25 mL of 50% ethanol)	*Eruca sativa* defatted seed meal (0.1~1 g/kg p.o)	administration of *E. sativa* seed (1 g/kg) promoted colon recovery from injury and decreased enteric gliosis	[52]
C57BL/6J mice, male, 6 weeks	water consisting of 2.5% (*w*/*v*) DSS	gavage with BSE (370 mg/kg·day) or with *Bifidobacterium longum* CCFM1206 (10^9^ CFU/mL)	combined treatment of *B. longum* CCFM1206 and BSE ameliorated DSS-induced colitis symptoms, mitigated colonic inflammatory levels and oxidative injury and restored the intestinal barrier	[53]
C57BL/6 mice, 8 weeks old	drinking 2.5% DSS for 9 days	diet supplemented with GRP (600 ppm) for 4 weeks	GRP attenuated body weight loss, DAI and colon shortening, maintained the colonic structure, inhibited inflammatory reactions and reduced colonic macrophage infiltration	[40]
C57BL/6 mice	drinking 4% DSS water for 5 days, acute colitis	pretreatment with 25 mg/kg·b.w. SFN per os for 7 days	SFN pretreatment significantly minimized body weight loss and DAI, extended colon lengths and relieved colon inflammation	[37]
C57BL/6 mice, male, 8 weeks old	drinking 3% DSS water	AITC in corn oil	AITC intervention showed less body weight loss, fewer colitis symptoms and longer colons, lessened the disruption of colonic histological structure and decreased mucosal inflammation	[54]
C57BL/6 mice, male, 8 weeks old	drinking 3% DSS water for 7 days	DIM	DIM significantly ameliorated the clinical symptoms and histological features, reduced inflammatory cell infiltration and suppressed the expression of pro-inflammatory cytokines and vascular endothelial growth factors	[55]
C57BL/6 mice, female, 6 weeks old	drinking 2.5% DSS water for 7 days	AITC (10 mg/kg/day) for 7 days	AITC could attenuate the severity of colitis through enhancing the intestinal barrier, including both TJ protein and mucin expression	[45]
BALB/cJ and C57BL/6 mice, female	50 μL intrarectal injections of 1 mg of TNBS in 50% ethanol	I3C (40 mg/kg in 0.05% DMSO/corn oil)	I3C repressed colonic inflammation and prevented microbial dysbiosis, increasing a group of butyrate-producing gram-positive bacteria, which was correlated with an increase in IL-22	[43]
C57BL/6 (18~22 g), male	drinking 2.5% DSS water	SFN intragastric administration 20 mg/kg·days for 2 weeks	SFN treatment increased body weight and colon length, decreased the colon damage scores and myeloperoxidase (MPO) activity, reversed DSS-induced gut microbiota dysbiosis and restored the abundance of *Butyricicoccus*	[29]
Sprague Dawley rats	intracolonic single administration of 2 mL of 4% acetic acid	SFN (15 mg/kg) by oral gavage daily for 2 weeks	SFN maintained the length and weight of the colon and improved morphological changes by improving antioxidant ability, elevating mitochondrial biogenesis and suppressing DNA polymerization	[42]
C57BL/6 mice, male	drinking 2% DSS water for 7 days	SFN (2.5, 5, 10 and 20 mg/kg body weight)	SFN treatment alleviated the changes in colon length, DAI scores and pathological damages, partially recovered gut microbiota disorder and enhanced the content of volatile fatty acids	[41]
C57BL/6JNifdc mice, male, 6–8 weeks old	2.5% DSS was gavaged for 7 days	SFN (20, 40, 10 mg/kg·days)	SFN effectively attenuated intestinal inflammation through skewing the switching from classically (M1) to alternatively (M2) activated phenotypes both in intestinal and bone marrow-derived macrophages, leading to changes in the inflammatory mediators	[38]
human colonic cancer cell lines HT-29 and Caco-2	treated with IL-1β (1 ng/mL) for 5 h	DIM	DIM mainly recovered the intestinal permeability of differentiated Caco-2 cells through increasing TJ protein expression and significantly enhanced the transepithelial electrical resistance of the cell monolayer	[44]
*Caenorhabditis* elegans, wild-type strain N2 and the mutant strain SS104	feeding with *Pseudomonas aeruginosa* PAO1	DIM	DIM relieved the damaged intestinal permeability and prolonged the lifespan of *C. elegans* fed *P. aeruginosa*	[44]

In conclusion, GLSs and their metabolites can effectively alleviate the IBD symptoms in in vivo and in vitro experiments. The intervention mechanisms are mainly intestinal microbiota regulation, antioxidant and anti-inflammatory effects, maintenance of the intestinal barrier and regulation of epigenetic mechanisms.

## 4. Mechanism of GLSs and Their Metabolites in Alleviating IBD

### 4.1. Regulation of Gut Microbiota

The dysbiosis of the gut microbiota is associated with the pathogenesis of IBD [32,56]. Individuals with intestinal inflammation have an imbalance in their gut microbiota, characterized by a decrease in bacterial diversity and alterations in the relative abundance of certain genera and species. A comprehensive reanalysis of fecal 16S rRNA amplicon sequencing data from 934 IBD patients and 1584 healthy individuals confirmed reduced alpha and beta diversity in IBD patients compared with healthy controls and identified 38 novel differential genera in CD and 28 novel genera in UC [57]. These lower-abundance genera in IBD patients included the *Lachnospiraceae* NK4A136 group, *Akkermansia*, *Faecalibacterium*, *Roseburia*, *Alistipes* and *Parabacteroides*, while these genera *Streptococcus*, *Enterococcus* and (*Ruminococcus*) *gnavus* group were more abundant in both CD and UC. The relative abundance of *Lactobacillus*, (*Eubacterium*) *xylanophilum* group and other *Muribaculaceae* also decreased in the DSS-treated mice [50].

It was observed that there is a decrease in the relative abundance of bacteria with anti-inflammatory activities belonging to *Clostridium* cluster IV and an increase in the proportion of bacteria with pro-inflammatory activities in IBD patients [56]. The increased pathogenic bacteria, particularly *Escherichia coli*, invade the intestinal epithelial cells and induces inflammatory responses [42].

#### 4.1.1. Regulation of Gut Microbiota Composition

**Figure 1:**
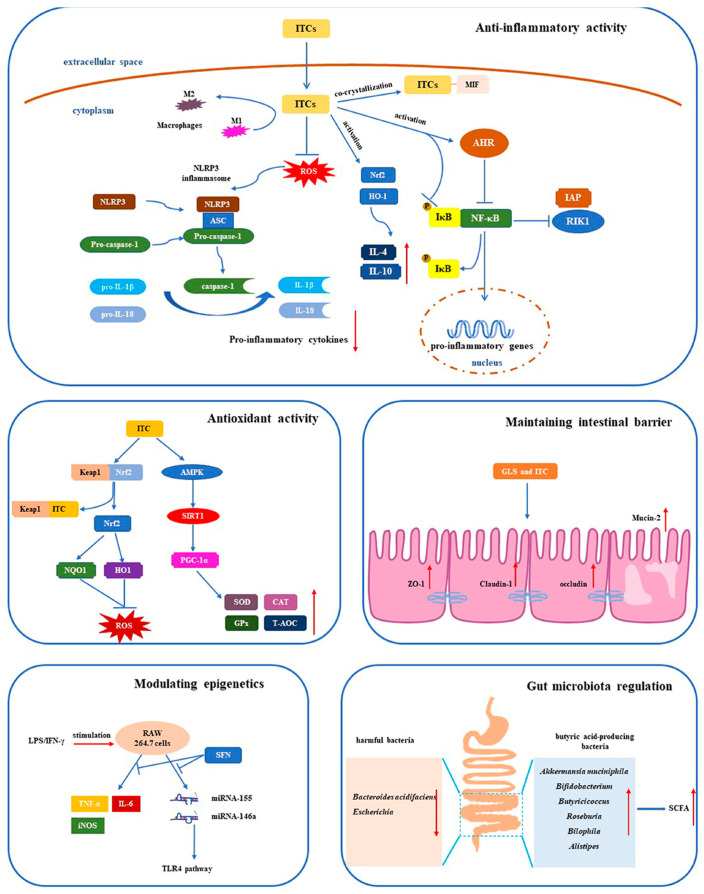
The attenuating mechanism of GLSs and metabolites on IBD through anti-inflammatory and antioxidant activity, maintaining the intestinal barrier, gut microbiota modulation and modulating epigenetics.

The gut microorganisms with myrosinase-like activities can hydrolyze GLSs, and the GLS metabolites in turn affect the gut microbiota [24]. Broccoli extract, which mainly contains polyphenols and GLSs, increases the proportion of beneficial bacteria such as *Bifidobacterium*, *Blautia*, *Coprococcus* and *Phascolarctobacterium*, and it reduces the harmful bacteria *Escherichia* in vitro, while GLS hydrolysis is remarkably associated with *Bilophila*, *Alistipes*, *Bifidobacterium*, etc. [58]. Red cabbage juice intervention improved the abundance of *Butyrivibrio*, *Roseburia*, *Ruminococcaceae*, *Acetatifactor muris*, *Rosburia* Sp. CAG:303 and *Dorea* Sp. 5–2 compared with the DSS group. In the case of *Clostridia*, it has been reported to produce butyrate [48]. Administration of steamed broccoli sprouts increased the richness and diversity of mice gut microbiota and altered the microbial community structure, resulting in increasing the relative abundance of *Firmicutes* and *Bifidobacterium* and the beneficial bacteria *Akkermansia muciniphila* (*Akkermansia*) while reducing *Bacteroides acidifaciens* [15,41]. *Bifidobacterium* produced short-chain fatty acids (SCFAs) to maintain the gut barrier, while *Bacteroides acidifaciens* degraded mucin and compromised the protective layer of intestinal epithelial cells, leading to colonic inflammation (Figure 1).

It has been demonstrated that GLSs and their metabolites can alter intestinal microbiota compositions in colitis models. SFN partially restored gut microbiota dysbiosis induced by DSS administration and increased the relative abundance of *Butyricicoccus*, synergistically exerting anti-inflammatory effects with *Butyricicoccus* [29]. I3C prevented TNBS-induced microbial dysbiosis, characterized by an increase in gram-negative bacteria, and selectively increased the proportion of the butyrate-producing gram-positive bacteria, especially *Roseburia* [43].

#### 4.1.2. Promoting the Production of SCFAs

SCFAs, a class of crucial metabolites of intestinal microbiota, show anti-inflammatory activity by binding and activating endogenous receptors, such as GPR41 and GPR43, to prevent immune reactions, improve the level of IL-10 and inhibit the production of IL17, thus protecting the intestinal barrier, suppressing excessive signaling of TLR and alleviating IBD symptoms [59,60,61].

Jaworska et al. demonstrated that there was an increase in the blood-to-stool ratio of SCFAs both in rats and pediatric patients with IBD, which was attributed to increased intestinal barrier permeability [62]. Generally, SCFA contents were decreased in IBD-related microbial dysbiosis, which is consistent with the depletion of SCFA producers such as *Faecalibacterium prausnitzii* and *Roseburia hominis* [63]. Although the levels of lactic acid, a SCFA precursor, were increased in CD and UC adult patients, microbial dysbiosis may prevent the conversion of lactic acid to butyric acid, leading to a decrease in SCFAs [63,64].

SFN administration promoted the production of SCFAs [41]. Treatment with I3C can increase the abundance of *Roseburia* (which produces butyrate) and the concentration of butyric acid [43]. Increased levels of butyric acid and isobutyric acid in mice can enhance the production of anti-inflammatory cytokines and regulate inflammation by reducing pro-inflammatory cytokines [65]. Butyric acid can be absorbed and utilized by intestinal epithelial cells, inhibiting neutrophil function, improving barrier function and reducing inflammatory responses [66]. Treatment with butyrate salts significantly improved intestinal inflammation in mice and protected the intestinal epithelial barrier [41]. Sodium butyrate can activate AMPK to induce mitophagy, alleviate hydrogen peroxide-induced oxidative stress and maintain the intestinal epithelial barrier’s integrity [67].

### 4.2. Antioxidant Activity

Although the underlying pathomechanisms of IBD have not yet been completely clarified, accumulating clinical and experimental evidence demonstrates that oxidative stress plays a critical role in the initiation and development of IBD [68]. Under physiological conditions, reactive oxygen species (ROS) and reactive nitrogen species (RNS) are produced in the intracellular organelles of intestinal cells. Once the antioxidant defense system is overwhelmed by high levels of ROS or RNS generation, excessive pro-oxidants attack the gastrointestinal mucosal layer and alter the inflammatory response [68], resulting in more cellular impairments, including DNA damage, protein aggregation and membrane dysfunction [69]. Certain gut microorganisms can also produce ROS directly, which further worsens intestinal inflammation [70].

GLSs and their metabolites have no direct antioxidant activity, but they can induce cell-protective enzymes, including various antioxidant enzymes, to exert indirect antioxidant effects [71]. SFN attenuates LPS-induced oxidative stress in Caco2 cells through improving the levels of superoxide dismutase, glutathione peroxidase, catalase and total antioxidative capacity, and it activates the AMPK/ SIRT1/PGC-1α pathway, which helps control the levels of ROS in mitochondria [72]. Cedrowski et al. found that SFN and erucin thermally decompose into sulfenic acids and methylsul finyl radicals, thus exhibiting antioxidant activity at high temperatures above 100 °C [73]. SFN strongly activates transcription factor EB (TFEB) to promote autophagic flux and lysosomal biogenesis, relieving oxidative stress [74].

GRP supplementation activated Nrf2, increased the levels of heme oxygenase-1 (HO-1) and xanthine oxidase and reduced the 8-hydroxydeoxyguanosine levels in DSS-induced colitis mice [40]. Nrf2 can reduce intestinal mucosal damage by inhibiting ROS production and enhancing the transcription of antioxidant target genes [75]. SFN treatment increased the Nrf2 levels in UC rats [42]. Nrf2 is negatively regulated by kelch-like ECH-associated protein 1 (Keap1), which promotes its degradation through the ubiquitin-proteasome pathway. Naturally occurring ITCs such as AITC, BITC and SFN can promote Nrf2 dissociating from Keap-1 [76,77]. Although there is little evidence that SFN can activate Nrf2 in humans [78], as an electrophile, SFN could react with the cysteine residues in Keap1 [79,80], forming a sulfenic acid-based adduct with Keap1 [81] and thereby rendering Nrf2 binding ineffective and inducing Nrf2 expression [82].

### 4.3. Anti-Inflammatory Activity

#### 4.3.1. Downregulating Inflammatory Mediators

**Inhibiting pro-inflammatory cytokines and maintaining anti-inflammatory cytokines.** Pro-inflammatory cytokines (tumor necrosis factor-alpha (TNF-α), IL-1, IL-6, IL-12, IL-17, IL-18, IL-21 and IL-23), especially IL-6, TNF-α and IL-1β, are associated with the initiation and progression of IBD [7,40,83], which result from recruited monocytes and activated macrophages. IL-10, transforming growth factor-beta (TGF-β) and other anti-inflammatory cytokines also contribute to the pathogenesis of IBD by reducing the inflammatory response [84].

GLSs and their metabolites could alleviate IBD symptoms by inhibiting the levels of pro-inflammatory cytokines and promoting the production of anti-inflammatory cytokines. GRP pretreatment downregulates inflammatory cytokines such as IL-1β, IL-18 and TNF-α [40]. I3C, the hydrolysate of GBS, is a natural ligand of the aryl hydrocarbon receptor (AHR) [85]. It has been reported that IBD patients have reduced levels of endogenous AHR ligands compared with healthy individuals. AHR ligands can alleviate colonic inflammation through AHR activation, making AHRs a potential target for IBD treatment [86]. Peng et al. demonstrated that AHR gene knockouts promote intestinal epithelial cell death and inflammation [87]. AHR activation can interact with inhibitors of apoptosis proteins (IAPs), namely ubiquitin ligase, to upregulate its expression, and IAPs can accelerate the degradation of receptor-interacting protein kinase 1 (R1PK1). R1PK1 is a key mediator of programmed cell death and inflammation [87]. AHRs activated by I3C reduce the expression of other pro-inflammatory cytokines, including IL-8, IL6-, IL-1β and TNF-α [87,88] while maintaining the level of anti-inflammatory cytokine IL-22 mRNA [89]. Philip B. Busbe et al. pointed out that I3C mainly induces IL-22 to alleviate inflammation [43]. DIM is also an AHR ligand which can reduce the IL-8 levels in human colon cells treated with IL-1β [44].

Lower concentrations (10~20 μM) of SFN suppress the expression of pro-inflammatory cytokines [90,91]. Treatment with SFN significantly reduced the concentrations of pro-inflammatory cytokines IL-6, IFN-γ and TNF-α in mice with DSS-induced colitis [15,41,90]. SFN also increased the content of the mediator STAT3, which is involved in colitis and colitis-associated colon cancer epithelial repair as well as inflammation processes [41], and it weakens the ability of LPS to induce the production of pro-inflammatory cytokines (IL-1β, IL-6, IL-8 and TNF-α) and pro-apoptotic caspases-3 and 9 [72]. The inhibition of IL-6 by SFN depends on the interference of Nrf2 with the binding of IL-6 gene transcription [39]. SFN can enhance the expression of Nrf2 and HO-1. The activation of Nrf2 and HO-1 enhances the production of anti-inflammatory cytokines, especially IL-4 and IL-10 [91,92]. Additionally, SFN can induce Nrf2 to stimulate immune responses and exert anti-inflammatory effects [93] as well as regulate the inflammatory enzyme iNOS to inhibit the production of NO [91].

***Interfering with M1 macrophage polarization.*** Activated specific immature macrophages in an inflamed alimentary tract secrete pro-inflammatory cytokines to aggravate severe reactions and barrier damage. Reconstruction of the barrier and elimination of inflammation are driven by the switch in polarization from the classically activated phenotype (M1) macrophages to the alternatingly activated phenotype (M2) macrophages [94]. The balance of M1 and M2 macrophages is associated with intestinal inflammation, which becomes more severe with an increase in M1 macrophages. LPS-activated mouse macrophages polarize to the M1 type by inducing pro-inflammatory marker proteins to alter their energy metabolism and reduce respiration [95]. SFN pretreatment successfully interferes with M1 polarization of LPS- and IFNγ-mediated THP-1-derived macrophages, producing high-energy cells with high glycolysis and high respiration and decreasing the expression levels of M1 (IL-23, CCR7, IL-1β, IL-6 and TNF-α) markers [94].

**Co-crystallization with MIF.** Macrophage migration inhibitory factor (MIF) is a pleiotropic cytokine which acts as a mediator of inflammation and innate immune response and has been associated with several conditions, including IBD [96,97]. It has been found that ITCs could co-crystallize with seven molecular targets, one of which is MIF [98]. ITCs inhibit the tautomerase activity of MIF through covalently binding its N-terminal cysteine, suggesting a potential pathway against inflammatory colitis.

**Attenuation of inflammasomes.** Inflammasomes are cytosolic multiprotein complexes of the innate immune system which assemble in response to invading pathogens or endogenous stress [99]. Activation of the inflammasome generates active caspase-1, which cleaves the precursor cytokines pro-IL-1β and pro-IL-18 into active IL-1β and IL-18 and induces pyroptosis [100]. The NLR family pyrin domain-containing 3 (NLRP3) inflammasome is composed of three parts: NLRP3, apoptosis-associated speck-like protein containing a CARD (ASC) and pro-caspase-1 [101].

Inflammasome activation was causatively linked to the development of chronic intestinal inflammation [102]. The expression of NLRP3, ASC and caspase-1 was significantly increased in a DSS-induced mouse colitis model, resulting in activation of related proteins and increases in the levels of IL-18 and IL-1β [103]. SFN administration reversed these changes to varying degrees, inhibiting NLRP3 activation and restoring IL-18 and IL-1β, thus decreasing intestinal inflammation. BITC could also inhibit inflammasome activation and IL-1β production in LPS- and ATP-stimulated THP-1 cells by suppressing the NF-κB pathway [104]. As a potential target for IBD treatment, the NLRP3 inflammasome needs more attention in the future.

#### 4.3.2. Inhibiting NF-κB

Nuclear factor-κB (NF-κB) is a key transcription activator factor in inflammation and plays a crucial role in inflammatory responses. It has been observed that constitutive activation of NF-κB inflames colon tissue in IBD patients [105]. Normally, NF-κB is bound to IκB proteins in the cytoplasm. Upon stimulation by various factors (including cytokines, growth factors, mitogens, microbial components or stress factors), IκB is phosphorylated, ubiquitinated and degraded, leading to the translocation of NF-κB into the nucleus, where it controls the transcription of pro-inflammatory biomarkers such as inducible nitric oxide synthase (iNOS), TNF-α and IL-1β, among others [35]. SFN could inhibit the phosphorylation of IκB, thereby suppressing the transcription of pro-inflammatory genes controlled by NF-κB. LPS activates NF-κB upon TLR4 signaling, resulting in the production of pro-inflammatory cytokines. SFN can inhibit TLR4 signaling [106], attenuate cell stimulation by LPS and significantly suppress the transcription and translation of iNOS, TNF-α and IL-1β.

I3C and DIM are effective inhibitors of NF-κB [107] and reduce T cell activation and pro-inflammatory cytokine production [108,109]. AHR, activated by I3C and DIM, inhibits the activation of NF-κB by directly binding to NF-κB, as well as promoting IAP activity and inhibiting the activation of R1PK1 [87]. Meanwhile, 6-MITC, a compound of Japanese horseradish, competitively inhibits glycogen synthase kinase-3β (GSK-3β) and NF-κB. GSK-3β is an important positive regulator of various pro-inflammatory cytokines and mediators, and its inhibition helps alleviate colonic inflammation [110]. In addition, Chang et al. pointed out that regulating the balance between Th17 and Treg is an effective strategy for the treatment and prevention of IBD. Th17 cells have pro-inflammatory effects, while Treg cells have anti-inflammatory effects, and I3C and DIM can inhibit Th17 cells and increase Treg activity [111].

### 4.4. Maintaining the Intestinal Barrier

The intestinal barrier protects the human body from external harmful factors. IBD is characterized by symptoms including decreased expression of tight junction (TJ) proteins and mucins and thus disruption of the intestinal epithelial barrier’s integrity [41,45]. The intestinal epithelium works as a barrier with a layer of physical and immunological defense which not only prevents the invasion of pathogens and microorganisms but also serves as a selective permeability membrane [45,50]. TJ proteins are non-classical transmembrane proteins which connect intestinal epithelial cells and thus maintain the semi-permeable properties of these cells [45].

DIM treatment enhanced the expression of TJ proteins (including occludin and ZO-1) and significantly restored intestinal permeability in differentiated Caco-2 cells treated with IL-1β [44]. AITC-regulated TJ proteins and mucin 2 alleviated DSS-induced intestinal injury and failure [45]. The administration of broccoli seed extract can upregulate mRNA levels and TJ protein expression, including claudin-1, occludin and ZO-1, being beneficial to the intestinal barrier [50]. Apart from countering oxidative stress, Nrf2-Keap1 has been reported as one of pathways which regulates TJ protein expression [75], and GLS can activate Nrf2, as we noted above.

### 4.5. Other Mechanisms

Epigenetic modifications include major DNA methylation, histone modifications and miRNA expression. Many studies have shown that GLS compounds could exert anticancer effects by modulating epigenetic mechanisms [112,113,114,115]. However, there is limited research on the alleviation of IBD through the modulation of epigenetic mechanisms.

MicroRNAs (miRNAs) are a group of single-stranded non-coding RNAs which regulate gene expression through targeting mRNA post-transcriptionally [116]. Imbalances in miRNAs in IBD patients mediate inflammation through various pathways [117], and miRNAs are also considered biomarkers for the diagnosis and prevention of IBD [118]. SFN and DIM can regulate different miRNA expressions through various pathways to protect the intestines [119]. For example, Saleh et al. found that SFN significantly downregulates the expression of miRNA-155 and miRNA-146a, which are key regulators of TLR4-mediated inflammatory response, at the epigenetic level [106].

## 5. Processing Strategy of GLS-Containing Cruciferous Vegetable

The metabolites of GLSs, including ITCs, indoles, ETNs and nitriles, vary greatly as they are influenced by several factors, which was well reviewed in previous articles [120]. According to our review on the effects of GLSs and metabolites on colitis, GLS metabolites such as SFN, AITC and I3C exert a beneficial effect on the colitis model. Studies demonstrated that ITCs are health-promoting GLS metabolites, whereas ETNs and nitriles have less beneficial health effects and even exert adverse effects [121]. The functional properties of ITCs can be attributed to the special structure of a —N—C—S group, whose highly electrophilic carbon atom can react with strong nucleophiles such as thiols or protein disulfide bonds in physiological conditions [122]. Therefore, the consumption of cruciferous vegetables with high bioactive compound contents is desirable.

### 5.1. Pretreatment

Processing treatments for cruciferous vegetables, such as cutting, blanching, freezing, heating and drying, affect GLS stability and metabolism in vitro and in the body, as well as their health benefits [123] (Table 3). Cutting vegetables leads to the enzymatic degradation of GLSs into ITC or nitriles at low pH levels or ETNs in the presence of epithiospecifier proteins (ESPs). Blanching pretreatment (30 s) followed by cooling treatment had no significant influence on the total GLS content [124].

### 5.2. Heat Treatment

Low-intensity heat treatment can increase ITC formation. For example, high-temperature conditions inhibit the activity of myrosinase and ESPs to an extent at 70 °C and 50 °C, respectively [30]. When heating above 60 °C, ESP activity decreases significantly, while myrosinase remains active, and thus bioactive ITC metabolites increase [121]. Once food processing raises the temperature of vegetables above 70 °C, this results in a low utilization rate of GLSs and a low SFN content [125]. Long-term heating led to thermally induced GLS breakdown and an increase in the formation of nitriles [121,126]. The chemical degradation product thioglucose reacted with the ITCs to further generate new degradation products, namely 4-hydroxy-3-(4-(methylsulfinyl) butyl) thiazolidine-2-thione and 3-alkyl-4-hydroxythiazolidine-2-thiones, during aqueous heating [127,128]. Neither health benefits nor toxic effects were found with 3-alkyl-4-hydroxythiazolidine-2-thiones up until now. Therefore, controlling the thermal intensity (temperature and time) can decrease the losses of GLSs. 

**Table 3:** Changes in GLSs and their metabolites after different processing.

Materials	GLSs Before Treatment	Processing Method	Detection Method	GLSs and Their Metabolites After Treatment	Ref.
broccoli cauliflower, white cabbages, red cabbages, Chinese cabbages, baby cabbages, white radish roots and red radish roots	Total GLS contents varied among vegetable type and could be ordered as follows: red radish root > broccoli > white cabbage > red cabbage > white radish root > baby cabbage > Chinese cabbage >cauliflower. The dominant GLS also depended on each vegetable type. For example, GRP accounted for 58.77% and 47.33% of the total GLS contents of broccoli and red cabbage, respectively.	blanching (30 s) and cooling at 2–4 °C for 5 min	HPLC quadrupole time of flight (QTOF)	blanching had little influence on the total GLS contents	[124]
		QF −/+ boiling (8 min)		Total GLS contents of QF groups were higher than other groups for each species. High treatment temperature in VD resulted in a low GLS content. Blanching VFD is suitable for GLS preservation. Boiling led to a further decrease in the GLS content. The order of total GLS contents for each species was QF-B > VFD-B > VD-B > OD-B. There were significant differences in the stability among different GLSs or cruciferous vegetables.	
		OD −/+boiling (8 min)			
		VD −/+ boiling (8 min)			
		VFD −/+ boiling (8 min)			
red cabbage	0.21 µmol/g FW of ITCs but 0.62 µmol/g FW ETNs and 0.17 µmol/g FW nitriles formed after homogenization	aqueous heat treatment at 100 °C	UHPLC, GC-MS	4-pentenenitrile ↓, 3-butenyl isothiocyanate ↑ and 1-cyano-3,4epithiobutane ↓ after short heat treatment (2–3 min), where nitriles accounted for 92% after 120 min of heat treatment	[121]
white cabbage	0.72 µmol/g FW of ITCs, 0.50 µmol/g FW of ETNs and 0.16 µmol/g of nitriles	aqueous heat treatment at 100 °C	UHPLC, GC-MS	3-butenenitrile ↓, 2-propenyl isothiocyanate ↑ and 1-cyano-2,3-epithiopropane ↓ after 3 min of heating, where nitriles accounted for 99.5% after 120 min of heat treatment	[121]
kohlrabi	0.85 µmol/g FW of nitriles, 0.34 µmol/g FW ITCs and no ETNs	aqueous heat treatment at 100 °C	UHPLC, GC-MS	4-(methylthio)pentanenitrile ↓ and 4-(methylthio)butyl isothiocyanate ↑ after 3 min of heating, where nitriles accounted for 99% after 120 min of heat treatment	[121]
red cabbage	the main GLSs were GRP, PRO, GIB and GBS	heating in boiling water	HPLC-DAD-ToF-MS, GC-MS	GLS degradation products included 3-butenenitrile, 5-(methylsulfinyl) pentanenitrile, indole-3-acetonitrile, 4-pentenenitrile, 3-phenylpropanenitrile and 1-cyano-2,3-epithiopropane. Formation of the corresponding nitriles increased over time, and ITCs did not accumulate in broths during boiling.	[126]
kohlrabi	the main GLSs were GER followed by glucoiberverin and smaller amounts of GIB, GRP and several indolic GLSs	heating in boiling water	HPLC-DAD-ToF-MS, GC-MS	Degradation products included 5-(methylthio) pentanenitrile, 4-(methylthio)butanenitrile, 4-(methylsulfinyl)butanenitrile, 3-(methylthio) propyl ITC and 4-(methylthio) butyl ITC. The relative ITC concentration steadily declined, and the corresponding nitriles increased over heating time.	[126]
red cabbage	high content of SIN, GIB and GRP as well as GBS and low amounts of GNA, glucoiberverin, GER and gluconasturtiin (GNS)	freshly prepared homogenates incubated for 1 h	UHPLC-DAD-TOF-MS, GC-MS	GLS hydrolysis product differed depending on the structure, mainly including corresponding ETNs, nitrile, amine and ITCs. SIN yielded high amounts of ETNs and amine, followed by ITC.	[122]
white cabbage	mainly SIN and GIB as well as GBS and lower amounts of GNA, glucoiberverin, GRP and GNS.	freshly prepared homogenates incubated for 1 h	UHPLC-DAD-TOF-MS, GC-MS	GLS hydrolysis product differed depending on their structure, mainly included corresponding ETN, nitrile, amine and ITCs. GIB and GRP yielded high amounts of nitrile, and ITC yielded and low levels of amine.	[122]
broccoli seeds	Aliphatic GLS content was 54.5−218.7 μmol/g fresh weight, accounting for >90% of the total GLS. The major GLSs were GRP and GER in 27 samples and PRO in 7 samples.	enzymatic degradation (ground and incubated at 25 °C for 2 h)	HPLC, GC-FID	ITC, nitrile and ETNs of SIN, GNA, GIB, GER and PRO, such as glucomesonitrile, SFN and butenylsulfuroside cyclonitrile	[129]
leaf mustard	content of SIN, GNA, PRO, GBS, 4-methoxyglucobrassicin, neoglucobrassicin and GNS ranges among the varieties	fermentation (20 °C for 4 days)	HPLC, GC-MS	three ITCs, three EPNs and two CNs, including SIN-ITC, GNA-ITC, GNS-ITC, SIN-EPN, GNA-EPN, Pro-EPN, SIN-CN and GNA-CN	[130]
broccoli seed extract	GRP	24 h of anaerobic fermentation with *B. longum*	HPLC, UHPLC Q Exactive MS	SFN, SFN−L-cysteine and erucin	[53]

Note: GC-MS = gas chromatograph mass spectrometer; LC-MS = liquid chromatograph mass spectrometer; HPLC = high-performance liquid chromatography; UHPLC-DAD-TOF-MS = ultra high-performance liquid chromatography diode array detection time-of-flight mass spectrometry; ↓ = decreasing; ↑ = increasing.

### 5.3. Drying and Freezing

The influence of the drying treatment on the GLS content varied widely, depending on whether the high temperature environment was adopted. Luo et al. found that the total GLS content was lower in the oven-drying (OD) and vacuum-drying (VD) groups than vacuum freeze-drying (VFD). The high temperature employed in OD and VD could facilitate the thermal degradation and hydrolysis of GLS by myrosinase [124]. Quick-freezing (QF) and VFD maintained both the GLS content and the activity of myrosinase well.

### 5.4. Fermentation

Apart from the plant myrosinase, microorganisms also can degrade GLSs. After fermenting at 20 °C for 4 days, the GLS metabolites included three ITCs, three EPNs, and two CNs in leaf mustard [130]. Fermentation of broccoli seed extract (BSE) by *B. longum* released SFN, sulforaphane−L-cysteine and erucin [53]. Consequently, the combined treatment of *B. longum* and BSE relieved the colitis symptoms and colonic inflammatory activity of the mice model, which could be attributed to the combined intervention improving the contents of SFN and sulforaphane−N-acetylcysteine in mice colons compared with the BSE intervention alone.

### 5.5. Other Observations

Moreover, the extent of GLS degradation during processing depends on the concentration of the vegetable broth and the vegetable source [121,126]. GLS degradation declined with a declining vegetable concentration after long boiling treatment [126]. According to the GLS stability, white and red cabbages are more suitable for processing than other cruciferous vegetables [124]. It was suggested that short cooking times are more appropriate for broccoli and red radish root for fresh eating.

## 6. Conclusions and Perspectives

This review focused on the physiological functions of GLSs and their metabolites in cruciferous vegetables, highlighting their roles in modulating microbiota, antioxidation, anti-inflammation, maintaining the intestinal barrier, and regulating epigenetics to alleviate IBD symptoms. Dietary preparations mainly composed of cruciferous vegetables appear to be a promising intervention strategy for IBD, especially UC.

However, some issues still need to be addressed in the future. The main aspects include the following. (1) There were differences in intestinal microbiota among the colitis experiments, resulting in difficulty in the analysis of colitis-related microbiota and suffering bias due to technical differences. Therefore, high-quality animal experiments and even clinical trials can be employed to obtain more consistent conclusions. (2) Due to the low bioavailability and poor stability of GLSs and their metabolites, micro- and nanocapsules could be applied to improve their stability and bioavailability, but there is limited research in this area thus far. (3) SFN, DIM, and other substances have been shown to regulate epigenetic mechanisms, and further studies are necessary. (4) Long-term intake of glucosinolates may cause gastrointestinal discomfort, and metabolites of glucosinolates such as nitriles have certain levels of toxicity. Thus, safety in consuming cruciferous vegetables needs to be emphasized. (5) Most studies on the intervention of IBD using GLSs and their metabolites have been conducted for animal models, and there are limited clinical trial data on human intestinal inflammation. More clinical trials are needed to obtain clinical scientific data and expand the application of GLSs and their metabolites to human health in the future.

## Figures and Tables

**Table 1:** GLS composition and contents in common brassica vegetables.

Classification of GLS	Compound Name	Glucosinolate Content (μmol/g)			
		Kale [20] *Brassica oleracea* L.	Broccoli [19] *B. oleracea* L. var. Italic)	Chinese Cabbage [21] *B. rapa* var. Glabra Rule)	Mustard [22] *B. juncea*
aliphatic GLS	glucoiberin (GIB)	4.78~5.42	0~0.878	N/A	N/A
	glucoberteroin	N/A	0.008~6.273	0.35~0.38	N/A
	glucoraphanin (GRP)	N/A	0.136~14.973	N/A	N/A
	progoitrin (PRO)	0.08~0.70	0~4.537	0.71~0.87	N/A
	sinigrin (SIN)	1.64~1.78	0~3.161	N/A	13.95~17.67
	glucoalyssin	N/A	0~2.728	1.11~1.19	0.45~0.53
	gluconapin (GNA)	N/A	N/A	0.23~0.27	0.12~0.18
percentage (%)		84.40~85.08%	27.78~48%	18.93~23.20%	71.56~90.69%
indole GLS	glucobrasscin (GBS)	0.98~1.04	0.103~27.690	3.94~4.36	0.43~0.59
	neoglucobrasscin	0.07~0.33	0.018~45.954	3.54~3.56	N/A
	glucobrassicanapin	N/A	N/A	0.85~0.91	0.20~0.28
	4-hydroxyglucobrassicin	N/A	0.014~3.289	0.08~0.12	0.23~0.35
	4-methoxyglucobrassicin	N/A	0.014~3.915	0.96~1.12	0.28~0.4
percentage (%)		13.74~14.64%	43.67~70.99%	73.90~86.22%	3.50~6.47%
aromatic GLS	gluconasturtiin (GNS)	N/A	0.004–0.441	N/A	0.22~0.34
	sinalbin	N/A	N/A	N/A	N/A
	glucotropaeolin	N/A	0–0.040	N/A	N/A
percentage (%)		0	0.42~1.33%	0	1.08~2.13%
total (μmol/g)		7.64~9.36	0.30~113.88	11.68~12.68	15.93~20.29

Note: Data in the table are measured by dry weight. N/A = undetected.

## Data Availability

The original contributions presented in this study are included in the article. Further inquiries can be directed toward the corresponding authors.
